# How to Choose a Good Marker to Analyze the Olive Germplasm (*Olea europaea* L.) and Derived Products

**DOI:** 10.3390/genes12101474

**Published:** 2021-09-23

**Authors:** Sara Sion, Michele Antonio Savoia, Susanna Gadaleta, Luciana Piarulli, Isa Mascio, Valentina Fanelli, Cinzia Montemurro, Monica Marilena Miazzi

**Affiliations:** 1 Department of Soil, Plant and Food Sciences, University of Bari “Aldo Moro”, 70126 Bari, Italy; sarasion86@gmail.com (S.S.); michele.savoia@uniba.it (M.A.S.); sanna14@hotmail.it (S.G.); mascioisa@gmail.com (I.M.); monicamarilena.miazzi@uniba.it (M.M.M.); 2 Spin Off dell’Università degli Studi Aldo Moro/SINAGRI S.r.l., 70126 Bari, Italy; luciana.piarulli@libero.it

**Keywords:** olive diversity, genotyping, traceability, molecular markers, morphological characterization

## Abstract

The olive tree (*Olea europaea* L.) is one of the most cultivated crops in the Mediterranean basin. Its economic importance is mainly due to the intense production of table olives and oil. Cultivated varieties are characterized by high morphological and genetic variability and present a large number of synonyms and homonyms. This necessitates the introduction of a rapid and accurate system for varietal identification. In the past, the recognition of olive cultivars was based solely on analysis of the morphological traits, however, these are highly influenced by environmental conditions. Therefore, over the years, several methods based on DNA analysis were developed, allowing a more accurate and reliable varietal identification. This review aims to investigate the evolving history of olive tree characterization approaches, starting from the earlier morphological methods to the latest technologies based on molecular markers, focusing on the main applications of each approach. Furthermore, we discuss the impact of the advent of next generation sequencing and the recent sequencing of the olive genome on the strategies used for the development of new molecular markers.

## 1. Introduction

Genetic diversity is a key resource for plant breeders aiming to develop cultivars with desirable characteristics such as yield enhancement and disease resistance. Over the centuries, plant diversity has been widely exploited, however, the expansion of intensive agriculture has led to the use of few productive genotypes and a progressive loss of genetic diversity. Recently, a growing awareness of the consequences of extreme climatic phenomena and the spread of new dangerous diseases has prompted efforts to save plant genetic resources as a reservoir of adaptive genes for future needs.

In the Mediterranean Basin, the olive tree (*Olea europaea ssp. europaea var. europaea*) is a fundamental crop, grown on 12 million hectares (95% of the world’s cultivated olive area) [1] with Spain and Italy as the major producers [2]. Extra virgin oil and table olives integrate the pillars of the Mediterranean diet and contribute to the success of this nutritional model. The worldwide appreciation of the Mediterranean diet is due to its valuable nutritional components, in particular antioxidants beneficial in reducing the risks of cardiovascular diseases [3].

The olive species (2n = 2x = 46) [4] belongs to the *Oleaceae* family, composed of 25 genera and 600 species growing both in the temperate and tropical regions of the world [5]. The species includes the subspecies *cuspidata*, *guanchica*, *cerasiformis*, *laperrinei*, *maroccana* and *europaea*, this last divided into the botanical varieties *europaea* and *sylvestris*, which correspond to the cultivated and wild olive (oleaster), respectively [6]. The two varieties are very similar, fully compatible via cross pollination, but the wild type oleaster, which grows spontaneously in the Mediterranean scrub, is characterized by thorny shrubs, smaller leaves and fruits with a lower oil content than the domesticated one [6]. The evolution of this plant probably started about six thousand years ago in the eastern coast of the Mediterranean, where it would have spread east to North Africa by Phoenicians, and through the Mediterranean Basin by the Greeks and Romans [7,8]. In accordance with this hypothesis, three main genetic pools were identified in the areas of the Eastern (Q1), Central (Q2) and Western (Q3) Mediterranean Basin, which would correspond to the crop’s centre of origin and diversification, following the introgression of local oleasters alleles [9,10,11,12].

Nowadays, about 2600 olive varieties are numbered worldwide, most concentrated in southern European countries Italy, Spain, France Tunisia, Algeria and Greece [13], but new local varieties and ecotypes are continuously detected in all the Mediterranean countries through the implementation of olive biodiversity recovery programs [14,15,16,17]. This germplasm is the result of long processes of adaptation to local needs and environmental conditions, and it preserves variability for many traits, such as low vigour, tolerance to extreme temperatures, salinity, diseases etc., that could be useful to face the challenges posed by the incumbent climatic changes and new pathogens spread. The last decades have seen the implementation of over 100 olive germplasm collections in all the Mediterranean countries, aiming to preserve the genetic diversity of the crop in a certain territory [18,19,20,21,22]. The correct identification of olive cultivars is the first step for their safeguarding, but in the olive, it is quite challenging due to the crop’s high degree of kinship, clonal variation, mixtures with international cultivars, exchange of plant material over the centuries [23,24,25,26]. The approaches used to study diversity in olive trees range from the accurate description of the bio-morphological and biochemical characters to the use of molecular biology techniques. In this review, we provide a global view on the strategies used for olive genotyping and diversity studies focusing on the advantages and disadvantages of each method, discussing also the impact of recent sequencing of the olive genome on the strategies used for studying olive diversity.

## 2. Morphological Markers

Morphological characterization of genetic resources refers to the process by which accessions are univocally identified through a systematic description of morphological characters. Traits must have high heritability and discriminating power at both the taxonomic and agronomic level, and they must be clearly distinguishable, easily recordable and expressed exactly and uniformly [27]. Species like Vitis vinifera take several advantages from ampelographic characterization [28], where the descriptors are well codified and recognized at the international level by the Organisation Internationale de la vigne et du vin: (OIV). The approaches used for the discrimination of olive cultivars based on morphological traits are shown in Table 1. The first attempt to characterize the olive tree is attributed, in the early 1940s of the 20th century, to Ciferri and Breviglieri [29] who used a standardized method for the morphological description of drupes, leaves, inflorescences, endocarps and other organs. The list of characters was further extended by Baldini and Scaramuzzi [30] and Damigella [31], but only in the 1980s, following the important development of the olive industry in Spain and Portugal, the olive germplasm description became decisive. Rallo et al. [32] extended the “Ciferri” list for the elaiographic description of olive varieties, leaving out the characters difficult to measure and more influenced by the environment, and including pictures of plant organs. In 1985, the International Union for the Protection of New Varieties of Plants (UPOV) [33] provided a reliable reference list of markers for a standardized data collection methodology which, together with that set up by the International Olive Oil Council (IOOC, Madrid, Spain, 1997), [34] is, today, the most used for primary characterization of olive varieties. In 2000, Barranco et al. [35] published the ‘World Catalogue of Olive Varieties’ including the plant passport data, and in 2004 Terral et al. [36] proposed an innovative approach based on the multidimensional morphometry of plant organs. More recently, Blazakis et al. [37] introduced a semi-automatic methodology for olive phenotyping, using image processing, and Gómez-Gálvez et al. [38] exploited high-resolution imagery for high-throughput analysis of olive canopy traits.

Morphological descriptors remain the basis for a first varietal identification, but their dependence on plant development stages, cultivation techniques and other environmental factors has led to their progressive use in combination with DNA analysis techniques, which have become essential in the evaluation of distribution and extension of genetic diversity in olive crops. Recently, thanks to the advantages of modern technologies, is possible to re-evaluate morphological charactersby phenomics approaches, which allow a more reliable scoring, a reduced influence of human observation with less time and money consumed [43,44]. Today, morphological markers resolved through automatic platforms have become particularly important in mapping population analyses, association studies and functional genetics approaches.

## 3. Molecular Markers

A molecular marker is a region of DNA associated with a specific location in the genome showing polymorphism of the nucleotide sequence in different individuals, due to insertion, deletion, point mutations, duplication and translocation [45]. Their use expanded enormously in the 1980s with the discovery of the Polymerase Chain Reaction (PCR), providing a rapid, highly informative and cost-effective tool for exploring plant genetic diversity. Over the years, different types of molecular markers were developed, such as Random Amplified Polymorphic DNA (RAPD), Restriction Fragment Length Polymorphism (RFLP), Amplified Fragment Length Polymorphism (AFLP), Simple Sequence Repeats (SSRs), Single-Nucleotide Polymorphism (SNP), each having peculiar advantages. In the olive, the applications of all these markers address different aspects of genetic studies, from cultivars identification to genetic maps construction, phylogenetic studies and food traceability [46,47,48]. Below, an extensive and comprehensive description of the main genetic markers used in olive genetic studies is presented.

### 3.1. RFLP Markers

Restriction Fragment Length Polymorphism was the first molecular marker developed and the only one based on hybridization. In this technique, DNA is cut by restriction enzymes in specific loci (recognition sites) resulting in a large number of fragments of various lengths due to the differences in DNA sequence in different individuals [49]. In the olive, RFLP markers were used in studies of phylogeny, taxonomy and analysis of variability of wild germplasm compared to the cultivated one [50,51,52,53], but they were the tool of choice also for linkage mapping, often in association with RAPD and AFLP markers [54,55,56,57,58]. After years of undisputed use, the RFLP technique suffered a decline following the introduction of markers based on the PCR, which is today at the base of most genetic diversity studies.

### 3.2. RAPD Markers

In Random Amplified Polymorphic DNA, the amplification of genomic DNA is achieved by PCR using a single, short random primer (10 nucleotides) which hybridizes similar sites at the opposite direction, producing amplicons dependent on the length and size of both the target genome and the primer [59]. They are characterized by simplicity and applicability, also to species little known at a genetic level, and for these reasons, they found wide application in the olive. RAPD markers were largely used alone or in association with other markers, for varieties characterization [18,60,61,62] and phylogenetic studies [7,63].

Unfortunately, the dark side of RAPD markers is a scarce reproducibility due to the low temperature of PCR annealing step and, consequently, a nonspecific amplification. These conditions favourited the predominance of other molecular markers.

### 3.3. AFLP Markers

The AFLP technique combines the digestion of target DNA with appropriate restriction enzymes with PCR. Matching the discriminating action of restriction enzymes and that of the polymerase amplification, they assure a good reproducibility and a high degree of polymorphism, allowing the simultaneous screening of a large number of loci, without any preliminary sequence knowledge [64]. In the olive, AFLPs have been used to study the variability and genetic relationships between cultivated varieties and wild forms [65,66], but also to solve identification problems in intra- and inter-varietal genetic diversity [67,68]. Used also in combination with other markers, such as inter-simple sequence repeat (ISSR), RAPD, sequence-characterized amplified region (SCAR) and SSR markers, they allowed the development of the first high-density linkage maps in the olive [55,69,70], paving the way to the identification of quantitative trait loci (QTLs) associated with important agronomic traits [71,72].

### 3.4. SCAR and CAPS Markers

Besides some above-mentioned PCR-based markers that have been frequently used in olive fingerprinting, other markers, derived from AFLP or RAPD markers, can be used. The Sequence-Tagged Sites (STS) is a short DNA sequence that has a single occurrence in the genome and whose base sequence and location are known. According to the detection method, it can be distinguished in Sequence Characterized Amplified Region (SCAR) or Cleaved Amplified Polymorphic Sequences (CAPS). The SCAR markers require the use of two locus-specific oligonucleotide primers derived from the nucleotide sequence, obtained by sequencing, of an amplified RAPD or AFLP fragment corresponding to a trait of interest. If the fragment of interest is amplified and treated with restriction enzymes revealing a polymorphism in the difference in length of restriction fragments, caused by SNPs or INDELs, these are called CAPS markers.

SCAR markers were successfully used to discriminated unequivocally varieties of the olive [73,74], but were also applied to oil analysis. In particular, SCARs developed from leaf DNA have proven not to be detectable in oil DNA, because they were too long or not abundant enough [75], for this reason, SCAR marker of chloroplast origin (CP-rpl16T) were isolated directly from a monovarietal oil AFLP profile [76].

### 3.5. ISSRs

ISSRs are DNA fragments between 100–3000 bp located between adjacent, oppositely oriented microsatellite regions, which can be amplified by using microsatellite core sequences as primers for PCR [77]. They are easy to handle, highly informative and repeatable, more reproducible than RAPDs due to the use of longer primers and higher annealing temperatures, and more manageable than AFLPs [78]. In olive, these markers were used in particular for phylogenetic analyses within the *O. europaea* subspecies [79] and olive germplasm characterization [80,81,82,83], also coupled with retrotransposon-based marker systems [84].

### 3.6. SSR Marker

SSRs or microsatellites are hypervariable short tandem repeat motifs of 1–6 nucleotides. The variation in the number of repetitions produces polymorphisms detectable by amplification with oligonucleotides complementary to the microsatellites flanking conserved regions [85]. Although their development requires a laborious procedure including the construction of a genomic library, cloning, sequencing and primer design, the use of SSRs, introduced in plant genetics in the early 90s [86], found immediately great application due to the advantages of being codominant, highly distributed throughout the genome, and highly reproducible with low quantity/quality DNA [87]. In the olive, microsatellite regions were sequenced in the early 2000s, and since the publication of specific primers [57,88,89], they have been increasingly used to dissect all aspects of a crop’s genetic diversity [90]. More polymorphic than RAPD and AFLP [91], they have often been used in combination with other molecular markers for synergistic enhancement, in particular, to discriminate related olive genotypes [92,93,94].

SSRs were used to investigate the origin of the crop [9,95] and the relationships between wild and domesticated olive. While different gene pools in the two varieties were found in southern Spain [96] and Sardinia [97], tight relationships between wild and domesticated olive were highlighted in olive germplasm from Northern Spain [98,99], Tunisia [100], Algeria [101,102], Israel [103], Morocco [104].

Microsatellites are also widely used for discriminating among different olive subspecies. In 2003, Rallo et al. [105] tested four microsatellites on cultivated olive along with different subspecies of Olea genus (subsp. *laperrinei*, *cuspidate* and *maroccana*) and other Olive taxa. Besnard et al. [10] analyzed a large population of cultivated olives, oleasters and Saharan olive forms (subsp. *Laperrinei*), evidencing a clear genetic differentiation between the two subspecies, but showing also, in the meantime, the presence of a few cases of admixture (two wild and three cultivated olives, namely “Dhokar”, “Ifri” and “Belluti”). The genetic variability between subsp. *europaea* and subsp. *cuspidate* was also studied through SSR markers. Hannachi et al. [106] analyzed genetic diversity among subsp. *europaea* (cultivated olive and oleaster) and subsp. *cuspidata*, identifying some hybrid plants. Hosseini-Mazinani et al. [21] compared microsatellite profiles of Mediterranean and Iranian cultivars with ecotypes and accessions of subsp. *cuspidata* underling a sharing of some alleles between Iranian and *cuspidata* samples.

In the last decades, the growing need to preserve the existing germplasm from genetic erosion resulted in the building of the large world germplasm collections, such as the World Olive Germplasm Banks (WOGB) at IFAPA (Cordoba, Spain) and Marrakech (Morocco), besides many small collections created in Europe [54,107,108,109], Northern America [110,111], Southern America [112], Australia [113], Northern Africa [114,115,116,117], the Middle East [118,119], the Balkan area [24,120,121], Iberia [122,123]. SSRs have been the marker of choice for genotyping these genetic resources.

In Italy, they allowed the unveiling of a vast and fragmented olive heritage, in accordance with Italy’s strong geographical and cultural articulation [15,124,125,126].

The advent of Next Generation Sequencing (NGS) strategies has, nowadays, led to the circumvention of the difficulties in isolating sequences from microsatellites [121,127]. The recent sequencing of the olive genome [128,129,130] has favoured the development of several new highly polymorphic SSRs distributed across the genome [131]. Therefore, the SSR markers currently available for the study of genetic characteristics and relationships of olive accessions are very high.

The increasing use of SSRs over the time highlighted limits linked to discrepancies between laboratories in the allele sizes assignment [132,133,134]. To overcome the drawback, a “consensus list” of alleles for a validated standard set of SSR markers with high power of discrimination, reproducibility (low peak stuttering), strong peak signal and absence of null alleles, was set up and became routine for olive genotyping [135,136]. Their application led to the implementation of public SSR-marker databases, such as the Italian “OLEA db” [137], the Olive Genetic Diversity Database (OGDD) relative to the Mediterranean germplasm [138], the Mendoza Argentina database [139] and the Algerian National Olive Germplasm Repository (ITAFV) [140].

Despite their many advantages, SSR markers efficiency could suffer from extremely degradated DNA and do not allow to distinguish clonal varieties of olive [141]. For this reason, the use of SNP markers is a valid aid in the study of traceability and authenticity of commercial olive oils. In fact, as shown in Chedid et al. [142], SNPs show a higher discriminatory capacity compared to SSR markers.

The combined use of SSR and SNP markers became widespread, in particular, in HRM (High Resolution Melting) analysis (SSR-HRM). This technique, based on differences in the melting temperature of PCR products, can magnify the polymorphism degree of microsatellites, and proved to be particularly effective in monovarietal oils authentication [142,143,144,145].

### 3.7. EST-SSR

Due to their high polymorphism, abundance and transferability, SSRs are the most used molecular markers for the characterization of the olive germplasm [27]. However, most published SSRs, consisting of dinucleotide repeats, showed several drawbacks due to the difficult discrimination between alleles [136]. For this reason, EST-SSR markers have been developed and used alone or in combination with SSR to dissect the genetic variability present in olive collections.

EST-SSRs, derived from expressed regions of the genome, have greater transferability between species than SSR markers and are localized within genes [22]. In addition, their variability may be related to phenotype [146]. However, EST-SSR may have less variability and polymorphism than standard SSRs, though sufficient for genetic population analysis and genotyping purposes [147].

EST-SSRs have been widely used in olive genetic evaluation [22,121,127]. The application of highly effective and informative markers, such as EST-SSR, allows the correct identification and diversity evaluation of olive accessions, making possible the adoption of the best conservation plan. The correct evaluation of genetic diversity is a crucial step to avoid redundancy in germplasm collections, decrease management costs and provide effective sources for genetic studies and breeding programs [22].

### 3.8. SNP Markers

A Single Nucleotide Polymorphisms is a small variation in the DNA sequence of different individuals, due to the substitution of a single nucleotide. These markers are codominant, abundant and uniformly distributed in genome, and their detection is highly reproducible among laboratories [125]. Since the first discovery of SNPs in olive, they found vast application worldwide in olive cultivars identification [107,148], genome mapping [149], phylogenetic studies [150,151]. Their use has undergone a huge increase thanks to the advances of next generation sequencing (NGS) technologies and the release to the public domain of the *O. europaea* whole-genome sequence by [128,129,130], making them very attractive for rapid processing of large collections and data management [152,153]. SNPs obtained by NGS technologies found an effective use in developing a high coverage saturated genetic linkage map as a prerequisite for a more efficient molecular breeding [154]. This aspect has seen the affirmation, in particular, of the genotyping by sequencing (GBS) technique [155] over other techniques, such as the more laborious and expensive Restriction site-associated DNA sequencing (RAD-seq) [156,157]. GBS consists of genome reduction through restriction enzymes, followed by fragments sequencing. The technique allows obtaining thousands of sequence-characterized SNP markers, providing a rapid, high-throughput and cost-effective tool to investigate plant genetic variability. Using GBS, Marchese et al. [158] and Ipek et al. [152] developed the first SNP-based high-density linkage maps in olive, while Belaj et al. [153], Taranto et al. [159] and D’Agostino et al. [12] shed light on the diversity of Italian olive cultivars and their geographical relationships, and Zhu et al. [160] focused on the close genetic relationships between Chinese and Mediterranean olive germplasm. More recently, Mariotti et al. [161] genotyped an F1 progeny derived from the cross between Leccino and Dolce Agogia cultivars by Restriction site associated DNA (RAD) markers sequencing, allowing the development of a high-density genetic map useful for trait mapping. 

SNP markers proved to be an excellent tool also for table olive and oil authenticity testing, being used in all the Mediterranean countries where these products have a substantial economic value [162].

**Figure 1:**
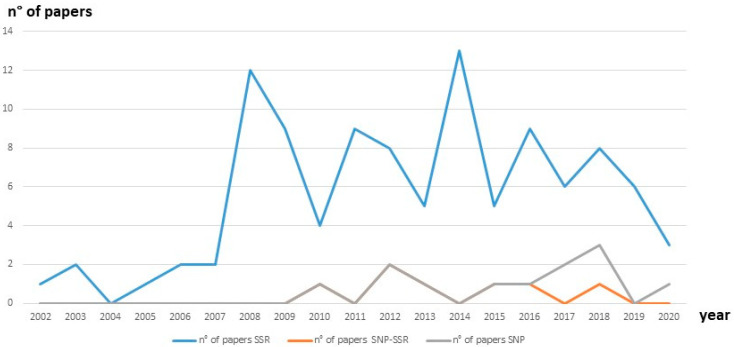
Use of the different types of markers for olive diversity studies during the last 20 years, based on the number of published papers using Scopus-indexed journal: SSR markers, the combined use of SSR–SNP markers and SNP markers. We can observe that the use of SSR for the cultivar identification increased by 2002 and it still is the most used, whereas the use of novel technologies and SNP markers is growing by 2017. By 2018, the combined use of SSR and SNP markers showed a powerful synergy in discriminating among highly similar cultivars, in resolving synonymies and homonymies and allowing a better cultivar identification, particularly in juvenile stages.

Figure 1 shows a comparative use of SSR and SNP markers for olive diversity studies over the past 20 years, highlighting how the use of SSR has increased in 2002 and how these markers are still the most widely used.

### 3.9. Molecular Markers Based on Transcriptome Analysis

Transcriptome analysis focuses on the variability of gene expression through a qualitative and quantitative description of the RNA transcripts differentially expressed by different individuals exposed to the same conditions [163], representing a powerful tool for studying the diversity in individuals. Through the years, transcriptomics has progressed from Northern blotting to RNA sequencing (RNA-seq) techniques, through real-time quantitative polymerase chain reaction (PCR) [164] and microarrays (from Affimetrix or Illumina) [165]. In the olive, the first transcriptomics studies addressed the identification of genes associated with important agronomic traits, such as drupes and reproductive organs development, fruit metabolism and phenolic content during ripening, through strategies such as suppression subtractive hybridization (SSH) and expressed sequence tags (ESTs) analysis [166,167,168,169,170]. SNP variations in EST were used to investigate the functional role of SNPs in specific agronomical performances [152], to uncover functional polymorphism in cultivated and wild olives [171], to infer phylogenies on the *Oleae* tribe [172] and for genetic identification of wild genotypes [173,174].

Again, the advent of next-generation sequencing (NGS) greatly enhanced transcriptome analysis, making it, in just a few years, a powerful method for rapidly identifying molecular markers associated with trait variation, leveraging both variations of the gene sequence and the variation of gene expression [175]. In particular, the use of RNA-seq was further consolidated by the lower cost per base pair, short time requirement and lack of subcloning process [176], becoming the preferred approach for transcriptomic studies in olive tree organs development [166,177,178,179], but especially for studying olive responses to biotic and abiotic stresses. Differential gene expression in relation to fruit total fatty acid content variations (palmitic, oleic and linoleic acid) was studied under adverse environmental conditions such as cold [180], salt stress [181,182], drought [183]. Gros-Balthazard et al. [184] used RNA seq to study the evolutionary story of the olive, comparing the differentially expressed genes in wild and cultivated accessions and individuating signatures of selection that support a major domestication event in the eastern part of the Mediterranean basin, followed by dispersion towards the west and subsequent admixture with western wild olives.

The transcriptomic approach, comparing the transcriptome profiles in the susceptible and tolerant olive cultivars, has been a huge help in identifying characters involved in the resistance to several biotic agents, helping marked assisted strategies for olive breeding. Grasso et al. [185] elucidated the mechanism of the inducible resistance to the olive fruit fly *Bactrocera oleae*, involving many metabolic pathways of oxidative stress responses, cellular structure, hormone signalling and primary and secondary metabolism. Leyva-Pérez et al. [186] and Serrano et al. [187] studied the olive’s mechanism of resistance to the soilborne fungus *Verticillium dahlia*, which still represents one of the most serious olive diseases due to the lack of effective disease control strategies. The analysis of differential transcriptomic root profiles in the tolerant and susceptible cultivars Frantoio and Picual, allowed the recognition of pathogenesis-related proteins involved in the lignification processes as possible markers for tolerant genotypes selection. A similar approach was used to study the fatal olive disease OQDS caused by the bacterium *Xylella fastidiosa* ssp. *pauca*. Comparisons between basal and infected transcriptomes in the xylem of the tolerant cultivar Leccino and susceptible varieties showed the involvement, in cv. Leccino tolerance, of the families of the *leucine-rich repeat receptor-like kinase* genes [188], ROS accumulation [189] and *Lignin* and *Cinnamoyl-CoA Reductase* genes [190].

### 3.10. Organelle Based and Ribosomal Markers

The use of mitochondrial and chloroplast DNA (mtDNA and cpDNA), due to their lack of introns, their haploid and uniparental inheritance, and their limited recombination, became popular in phylogenetics and population genetic studies. Nowadays, the availability of complete organelle genome sequences in many genera makes them suitable to access the variability of genomes at low taxonomic levels.

Variation in ribosomal and cytoplasmic non-coding DNA, like the internal transcribed spacer (ITS) and intergenic spacer (IGS), is largely used for phylogenetic studies. The high nucleotide variability, due to the lack of strict mechanisms of conservation of these sequences, promotes the availability of these markers for evolutionary purposes. Variations in these regions can be detected through direct sequencing or digestion of amplified sequences with restriction enzymes [191].

These organelle-based markers have been used in the olive for different purposes. Specific chloroplast and mitochondrial RFLP polymorphisms have been used to detect male sterility in several olive cultivars [192]. Chloroplast markers were analyzed in oleasters and cultivated forms of the Mediterranean basin, highlighting the chlorotype-specific marker ofthe Eastern basin in several cultivated forms [193]. In addition, mitochondrial RFLP analysis confirmed a clear genetic distinction between wild olives from the Eastern and Western parts of the Mediterranean basin [53,194]. An intergenic spacer of the mitochondrial genome was also used to test the effect of prolonged vegetative multiplication in the maintenance of mitochondrial homoplasmy. This study allowed confirming the role of sexual reproduction in the maintenance of mitochondrial homoplasmy [195]. Intrieri et al. [196] suggested an identification protocol for olive cultivars based on the amplification and subsequent sequencing of the chloroplast trnT-trnD intergenic spacer, revealing the usefulness of chloroplastic markers for cultivar identification and oil traceability.

Even ribosomal RNA markers have been proven to be useful markers for studying phylogenetic relationships because of their universal nature and the presence of conserved and variable domains. *O. europaea* genus has been described by analyzing polymorphisms in the internal transcribed spacers (ITS1 and ITS2) of the nuclear ribosomal genes 18S, 5.8S and 26S. The use of these markers allowed the reconstruction of the colonisation history of *O. europea L*. in the Macaronesian islands [63] and to study the genetic differentiation of cultivated olive from its wild relatives [55]. Moreover, ribosomal DNA markers were used to analyse the structure of olive tree populations belonging to subspp. *europaea* and *cuspidata* from Australia and Hawaii [197], and using both ribosomal and cytoplasmic sequences, Besnard et al. [198] revised the correlations within the *Oleaceae* family. More recently, other authors have analyzed chloroplast genome variation to perform *O. europaea L*. evolutionary analysis [199,200], confirming the wide potential of these markers.

### 3.11. The Molecular Markers Used for Olive Oil Traceability Purposes

The use of molecular markers for traceability has become of considerable importance following the increased economic value of olive oil, and the introduction of the European product certification “PDO” (Protected Designation of Origin). This trademark, certifying the place of origin and processing of the raw material, provides a guarantee to all the players in the oil supply chain, protecting the products from abuse and imitations (Community Regulation 2081/92). Moreover, the increase in the economic value of extra virgin olive oil (EVOO) and the appreciation of its organoleptic properties and health benefits by consumers lead fraudsters to blend low-cost poor-quality oil with EVOO to get economic benefits. Therefore, fast, precise, accurate and up-to-date analytical methods are required to detect adulteration of EVOO. Olive oil authentication, traditionally assessed by chemical approaches, analyzing the content of metabolites such as fatty acids, volatile compounds and tocopherols, in recent years has seen prevailing DNA-based methods [201]. Different molecular markers have been used to trace olive oil products.

The AFLP technique was optimised for fragmented DNA for oils traceability studies, overcoming the PCR’s inhibition problems due to the presence of high amounts of phenolic compounds [94]. Nevertheless, the most used molecular marker in olive oil traceability is SSR [202]. SSRs were efficiently used to authenticate and trace olive varieties in monovarietal and polyvarietal oils [201]. Besides, SNP was also revealed to be highly reliable in EVO oil traceability [48]. Recently, a (CAPS) assay set up for SNP analysis that alters the restriction enzyme recognition motifs was also developed. This method was used for detecting olive oil admixtures in a binary blend of oils prepared at a laboratory scale at different ratios. The restriction digestion-based SNP genotyping was found to produce highly reproducible results thanks to the fact that genotyping is based on the observation of the digestion patterns and does not involve any fluorescence signal measurements or fragment size comparisons [203].

Like many food matrices, DNA in olive oil can be highly degraded and present in very low quantities [201]. For this reason, the identification of the varieties present in commercial olive oils requires adequate DNA extraction protocols. Over the years, many DNA extraction protocols from oil based on CTAB and/or hexane have been developed [204,205] showing different results in terms of quality and quantity of recovered DNA. Recently, Piarulli et al. [48] set up an efficient method for DNA extraction from filtered EVO oil suitable for molecular markers-based traceability purposes.

Table 2 summarizes the main studies contributing to the development of different molecular markers in the olive.

### 3.12. Tips for Choosing the Best Molecular Marker to Dissect the Olive Diversity

The various molecular markers differ in their main characteristics, including the degree of polymorphism and type of distribution throughout the genome. Table 3 presents a list of the most commonly used molecular markers and their main advantages and limitations. The choice of the best molecular marker mostly depends on the objective of the study, operator’s experience, equipment availability and analysis cost.

In olive, AFLP and ISSR were used for the construction of genetic maps, phylogenetic analyses and intra- and inter-varietal genetic diversity studies [69,70,80]. In the past, amplicons were run on a polyacrylamide gel, making the technique quite laborious. Today, the detection of amplicons is mainly performed through capillary electrophoresis. Although they are highly abundant in the genome and they do not require prior sequence information, nowadays, their use is limited due to the spread of more reliable and informative SSR and SNP which are suitable for automation.

The sequencing of the olive genome boosted the spread of SSR and SNP markers. Their high level of polymorphism and reproducibility make them the markers of choice for most genetic diversity studies. Moreover, SSR and SNP markers can be detected on a very small portion of DNA, which, in the case of highly fragmented DNA such as those extracted by olive oil, may constitute an important advantage [201]. SSR markers are the most used markers in olive genetic diversity assessment. The most common approach for the fragment size evaluation is based on the use of capillary electrophoresis. Nevertheless, the analysis of amplicons by high resolution melting (HRM) assay was demonstrated to be highly effective. In the last years, the low cost and ease of application of the SSR/HRM technique has made this approach widespread [142,206,207].

SNPs are the most abundant markers and their diallelic nature allows a reduced error rate in allele calling compared with other molecular markers, making analysis based on SNPs highly reliable. Moreover, SNP identification is suitable for different detection methods such as CAPS assays, HRM and sequencing techniques. The CAPS assay is the easiest method to detect the SNP variant, however, its limited reliability has made this approach rarely currently used. Detection of SNP through HRM is much more widespread since it was demonstrated to be a fast, simple and reproducible method [142,208]. The most reliable approach is that based on the sequencing of the genomic region encompassing the SNP. Although this approach requires specialized personnel and has higher costs compared to HRM, it is quite often used [48].

The recent technical advances in sequencing methods that occurred have made SNP markers a selection tool in olive genetics study, allowing the development of high-density genetic maps and the study of geographical relationships [12,152,158]. Due to the several advantages of these markers, their use is expected to keep growing in the future.

## 4. Conclusions

During the last years, studies on molecular markers, genomics and transcriptomics of olive trees have rapidly increased, due to technical advances and innovative solutions. The application of molecular markers is widely recognized as a powerful tool to accelerate breeding programs (MAS Markers Assisted Selection), to perform cultivar identification, to investigate the genetic relationship and to trace raw material and processed foods. The ideal marker is an abstract concept because the right choice depends on several factors, such as money availability, instruments, people and the question to answer, to solve the experimental problem. Fortunately, the availability of tools is quite large, and, by a deep evaluation of advantages and disadvantages, it is possible to choose the best marker to apply in every experiment. This review will contribute to the continuation of the alternatives present in the olive sector and help researchers who will approach olive studies for the first time.

## Figures and Tables

**Table 1:** The approaches used for the description and discrimination of olive cultivars using morphological traits.

Year	Descriptors	References
1940	Fruits, leaves, inflorescences and endocarp	[29]
1950	Leaves, drupes and stones	[30,31]
1960		
1970		
1980	Whole plant, fruiting branches, leaves, inflorescences, fruits and endocarp	[32,33]
1990	Changes to the list of UPOV descriptors and addition of agronomic characters	[39,40]
2000	Plant passport data, qualitative and quantitative morphological descriptors	[41,42]
		[35,36]
2010	Morpho-geometric analysis on existing and fossil olive stones	[37]
	Analysis and image processing of leaves, fruits and endocarp	
2020	High resolution imagery for analysis of olive canopy traits	[38]

**Table 2:** Summary of the principal DNA-based molecular markers applied in *Olea europaea* studies.

Molecular Marker	Developers	Application in *Olea europea* L.	References
RFLP	Williams et al., 1989	Wild and cultivated olea variability	[49]
		Phylogenetic studies	[50,51,52,53]
		Genetic maps	[54,55,56,57,58]
		Development of organelle-based markers	[53,192,193,194]
RAPD	Williams et al., 1990	DNA fingerprinting of cultivars	[18,60,61,62]
		Phylogenetic studies	[7,63]
AFLP	Vos et al., 1995	DNA fingerprinting of cultivars	[65,66]
		Phylogenetic studies	[67,68]
		Construction of linkage map	[55,69,70]
		QTL identification	[71,72]
SCAR and CAPS	Paran and Michelmore, 1993	DNA fingerprinting of cultivars	[73,74]
		Cultivar traceability in olive oil	[76,203]
ISSR	Zietkiewicz et al., 1994	Phylogenetic studies	[79]
		Germplasm characterization	[80,81,82,83]
SSR	Morgante and Olivieri, 1993	Phylogenetic studies	[9,95,96,97,98,99,100,101,102,103,104]
		Subspecies analysis	[10,21,105,106]
		DNA fingerprinting of cultivars	[54,107,108,109,110,111,112,113,114,115,116,117,118,119,120,121,122,123]
		Cultivar traceability in olive oil	[142,143,144,145,201]
EST-SSR		Germplasm characterization	[22,121,127]
SNP	Wang et al., 1998	Cultivar identification	[12,107,148,153,159]
		Genetic maps	[149,152,158]
		Phylogenetic studies	[150,151,160]
		Cultivar traceability in olive oil	[48]

**Table 3:** List of the most commonly used molecular markers and their main advantages and limitations.

Marker	Detection System	Advantages	Disadvantages	* Cost Per Sample
AFLP	Capillary electrophoresis	High genomic abundance High polymorphism No sequence information is required	Laboriousness of the technique Dominant markers Expensive	50 euro
ISSR	Capillary electrophoresis	High genomic abundance No sequence information is required	Slightly informative Dominant markers	10 euro
SSR	Capillary electrophoresis	High polymorphism	Reduced genomic abundance	10 euro
	High Resolution Melting	High polymorphism Low cost	Reduced genomic abundance Require optimization	5 euro
SNP	High Resolution Melting	High genomic abundance Low cost	Require optimization	5 euro
	CAPS	High genomic abundance Easy to perform	Expensive Low reliability	20 euro
	Sequencing	High genomic abundance High reliability	Specialized personnel	15 euro

* costs per sample, are referred to a single marker and analyses in a small research laboratory providing all in house works.

## Data Availability

Not applicable.
